# Supplementary material for: Dietary Management Tools Improve the Dietary Skills of Patients with T2DM in Communities
Source: Nutrients. 2022 Oct 23;14(21):4453. doi: 10.3390/nu14214453 (PMC9654010; doi:10.3390/nu14214453)
Supplement: Supplementary file 1 [file nutrients-14-04453-s001.zip › nutrients-1929075-supplementary.pdf]

Table S1. The BIC Results of the Food Guidebook Usage Tendencies.

Group	BIC	Percentage of the smallest group
1	-2263.1	-
2	-2253.8	27.1%
3	-2193.9	23.5%
4	-2173.4	10.4%
5	-2188.5	5.4%

Table S2. Fit Parameter Estimates for the Four Food Guidebook Usage Subgroups.

Subgroups	Parameter	Parameter estimation	S.E.	t	P
1	Intercept	-6.293	1.645	-3.825	<0.001
	Linear	3.938	0.870	4.525	<0.001
	Quadratic	-0.514	0.130	-3.926	<0.001
	Cubic	0.020	0.005	3.483	<0.001
2	Intercept	1.243	0.036	33.705	<0.001
3	Intercept	5.366	0.230	23.277	<0.001
	Linear	-0.782	0.073	-10.641	<0.001
	Quadratic	0.037	0.004	7.750	<0.001
4	Intercept	4.059	0.274	14.802	<0.001
	Linear	-0.213	0.084	-2.511	0.012
	Quadratic	0.012	0.005	2.181	0.029

Table S3. The BIC Results of the Dish Set Usage Tendencies.

Group	BIC	Percentage of the smallest group
1	-2581.8	-
2	-2390.1	32.1%
3	-2347.4	17.3%
4	-2341.3	6.8%
5	-2416.9	7.2%

Table S4. Fit Parameter Estimates for the Three Dish Set Usage Subgroups.

Subgroups	Parameter	Parameter estimation	S.E.	t	P
1	Intercept	-0.145	0.054	-2.673	0.008
	Linear	0.658	0.258	2.546	0.011
	Quadratic	-0.095	0.0381	-2.501	0.012
	Cubic	0.004	0.002	2.444	0.014
2	Intercept	6.604	0.363	18.162	<0.001
	Linear	-1.125	0.117	-9.583	<0.001
	Quadratic	0.058	0.007	7.412	<0.001
3	Intercept	3.539	0.120	29.369	<0.001
	Linear	-0.030	0.015	-2.010	0.044
